# Trauma treatment across Europe: where do we stand now from a perspective of seven countries

**DOI:** 10.3402/ejpt.v7.29450

**Published:** 2016-03-18

**Authors:** Evaldas Kazlauskas, Jana Javakhishvilli, Mariel Meewisse, Dorota Merecz-Kot, Vedat Şar, Ingo Schäfer, Ulrich Schnyder, Berthold P. R. Gersons

**Affiliations:** 1 Department of Clinical and Organizational Psychology, Vilnius University, Vilnius, Lithuania; 2 Institute of Addictology, Ilia State University, Tbilisi, Georgia; 3 Specialty Center for Complex Trauma and Personality Disorders, GGZ Noord-Holland Noord, Heerhugowaard, The Netherlands; 4 Nofer Institute of Occupational Medicine, Lodz, Poland; 5 Department of Psychiatry, Koç University School of Medicine (KUSOM), Istanbul, Turkey; 6 Department of Psychiatry and Psychotherapy, University Medical Centre Hamburg-Eppendorf, Hamburg, Germany; 7 Department of Psychiatry and Psychotherapy, University Hospital Zurich, University of Zurich, Zürich, Switzerland; 8 Arq Psychotrauma Expert Group, Diemen, The Netherlands; 9 Department of Psychiatry, Academic Medical Center, Amsterdam, The Netherlands

**Keywords:** Trauma, treatment, PTSD, Europe, Georgia, Germany, Lithuania, Netherlands, Poland, Switzerland, Turkey

## Abstract

There is a lack of knowledge about the state of affairs of the trauma treatments in Europe. To start to fill in this gap, key persons from seven European countries—Georgia, Germany, Lithuania, the Netherlands, Poland, Switzerland, and Turkey—accepted the invitation to give their expert opinion on the state of affairs in their country at an invited panel discussion at the XIV 2015 ESTSS Conference in Vilnius, Lithuania. Brief reports from the seven countries reveal significant diversities among different European countries in terms of awareness of health problems related to trauma, the availability of trauma treatments, and treatment approaches. Political and economic differences across the European countries contribute to the diversities in the developments of trauma treatments. European national psychotrauma societies are active in establishing training curricula and dissemination of trauma-focused treatments. Despite the growing acknowledgment of trauma and dissemination of trauma-focused treatments, there is a lack of Europe-wide policies to ensure availability of trauma treatment in Europe for trauma survivors. The need for more detailed analysis of trauma treatment in all European countries and development of European-level trauma-informed health care policies is outlined.

Effective and evidence-based trauma-focused treatments have been developed and disseminated during the last few decades; however, little is known about the actual state of affairs of trauma practice in Europe. To start to fill in this gap, key persons from seven European countries—Georgia (Jana Javakhishvilli), Lithuania (Evaldas Kazlauskas), the Netherlands (Mariel Meewisse), Poland (Dorota Merecz-Kot), Turkey (Vedat Şar), Germany (Ingo Schäfer), and Switzerland (Ulrich Schnyder)—accepted the invitation to give their expert opinion on the state of affairs in their country at an invited panel discussion at the XIV 2015 ESTSS Conference in Vilnius, Lithuania. The panel was chaired by Berthold Gersons from the Netherlands. The discussion was organized around the following topics: the spread and availability of the trauma treatments in the country; the variety of treatment modalities available in the country; identification of the groups with posttraumatic problems that need treatment in the country; and how aware is the mental health field in the country about the impact of trauma in mental health problems and disorders, and in treatment? Descriptions of the situation in the seven European countries about the trauma treatment presented below are based on the expert opinions of the panel members and only partly answer some of the questions.

## The situation in Georgia

Demand for trauma care and disaster management is growing in Georgia. Since regaining independence in the early 1990s, Georgia experienced a series of armed conflicts culminating in a war with Russia in 2008. The conflicts resulted in more than 300,000 internally displaced persons (IDP) and other war-affected populations in need of psychosocial care, who constitute the key target risk groups for trauma care (Chikovani et al., 2015; Javakhishvili, 2014). Factors that contribute to the rapid growth of the psychotraumatology in Georgia are geopolitical reasons, the willingness of the local professional community to respond, and relevant projects-based international support and networking with ESTSS. Since 2007, the Georgian Society of Psychotrauma (GSP) is functional and focused at building a professional capacity and lobbying for putting in place evidence-based policies and practices in the field of trauma care. To assure trauma-informed mental health services and primary health care, a number of trainings and workshops were implemented during the last decade with the relevant professionals and decision makers. However, a consistent national strategy has still to be developed in this regard. In 2012, the Psychotraumatology masters’ program was introduced at Ilia State University.

The trauma-focused cognitive behavioral therapy (TF-CBT) and brief eclectic psychotherapy for posttraumatic stress disorder (BEPP) are the most widespread methods of trauma-focused treatment in the country. In spite of the fact that universal coverage of health care was introduced in 2013, psychotrauma is still not on the list of mental health conditions covered by medical insurance. The efforts should be continued to assure better access to mental health care for the most vulnerable traumatized groups in the country.

## The situation in Germany

In the German health care system, psychotherapists are psychologists or physicians with additional training in psychotherapy. Most of them offer CBT or psychodynamic/psychoanalytic therapies, as these modalities are covered by the mandatory health care insurance. Despite some postgraduate knowledge on trauma treatment, many therapists do not feel competent to deal with traumatized patients (Bergmann, 2011). As a result, many patients still face substantial problems to find adequate help (Bundespsychotherapeutenkammer, 2011). Therefore, the German-speaking Society for Psychotraumatology (DeGPT) has developed and implemented an additional trauma curriculum for psychotherapists, offered by about 30 institutes in the country. Curricula for child and adolescent therapists as well as other professionals in the educational and mental health field have followed.

Some progress has been made in the field of childhood sexual abuse. Reports about systematic abuse in institutions in 2010 have stimulated German ministries to install an independent commissioner into childhood sexual abuse (Bergmann, 2011). Several initiatives to fight childhood sexual abuse and improve the situation of victims followed, including substantial funding of research, preventive activities, and the possibility of financial compensation. Traumatized refugees are another important group, which is attracting increasing attention from the trauma field. While the lack of adequate help for this group has been evident for a long time, it becomes an increasing challenge and will obviously be one for all of Europe.

## The situation in Lithuania

Trauma research and practice emerged soon after the restoration of independence of Lithuania in 1990. The specific context with thousands of survivors of political oppression inspired research on the effects of political violence (Kazlauskas & Zelviene, 2016). Lithuanian suicide rates are three times higher than the EU average, and professionals are developing support programs for the families after the suicide of a family member. There is a growing interest among the police and military about trauma.

Courses about trauma and trauma-focused treatments are now included into post-diploma psychotherapy trainings and clinical psychology training curricula in the universities. Psychological treatment of trauma in Lithuania is largely influenced by psychoanalysis, as the psychodynamic approach currently dominates in the country. Existential therapy and CBT trainings expanded a picture of available trauma-informed treatments during the last two decades. BEPP was introduced in Lithuania in 2011. The Lithuanian Society of Traumatic Stress Studies was established in 2013 and is taking an active role in organizing trainings for professionals.

Despite these positive developments, there is not enough understanding of trauma and stress-related disorders in Lithuania. The number of professionals trained in trauma-focused psychotherapies is low. There is a national network of primary mental health care centers and inpatient clinics, and mental health services are covered by the National Health Care Insurance (NHCI). However, access to evidence-based trauma-focused treatments is very limited. The first treatment offered in the public health care system for mental health disorders, including posttraumatic stress disorder (PTSD), is usually a psychopharmaceutic treatment. Trauma survivors need to address professionals in private practice or NGOs if they want to get trauma-focused psychotherapy. Unfortunately, these services are then not covered by the NHCI.

## The situation in the Netherlands

The scientific interest in psychotrauma in the Netherlands as well as health care facilities has increased exponentially during the last three decades (Vermetten & Olff, 2013). The costs for treatment are largely covered by the Dutch mandatory health insurance. The government pays substantial attention to disasters, whereas the media writes daily to weekly about the effects of trauma and their sequelae. This enables general practitioners, occupational doctors, and other health care professionals to recognize the link between trauma and symptoms, and refer the patients to adequate care. Traditional stabilizing interventions in the treatment of PTSD are increasingly replaced by trauma-focused interventions. Eye movement desensitization and reprocessing (EMDR), in particular, is widespread, and TF-CBT, narrative exposure treatment (NET), and BEPP are disseminated as well. Unfortunately, the courses in these interventions are seldom included in the obligatory post-diploma educational programs for psychologist and psychiatrist in their clinical training.

PTSD due to events in adulthood in fairly well functioning patients seems well treated and special services and care is available for the military and police. However, there is still a lack of specialist knowledge, and accessibility to health care is hindered due to long waiting lists. Specifically, traumatized refugees and the more complex cases of PTSD related to childhood abuse still form a challenge in health care services.

## The situation in Poland

Although the research interest in psychotraumatology has been presented for years in Poland (e.g., Lis-Turlejska, 2008), there has been a lack of well-educated practitioners (Witteveen et al., 2012). In 2007, the Polish Society of Traumatic Stress Studies (PTBST) was established, and in 2010, the first regular postgraduate studies in psychotraumatology at the University of Social Sciences in Warsaw have started. Since 2015, psychotraumatologist has been officially recognized as a health profession. Before 2007, no coherent training on psychotraumatology was offered. Nowadays, evidence-based psychotherapy training (CBT, EMDR, BEPP) and training based on a psychodynamic approach are regularly organized in Poland. However, the issue of certification and standardization of training is not well regulated legally, thus the quality of some training might be questionable.

The number of well-trained trauma therapists is small. They usually work outside the health insurance system; hence, the costs of psychotherapy are covered directly by clients. Medical doctors are not generally familiar with the mental health problems of those traumatized. The first choice treatment offered by GPs for trauma survivors is pharmacotherapy. Due to the small number of professionals working with trauma in Poland there is no real competition between therapists representing different therapy modalities, and clients do not give much attention to the treatment methods.

Thus, the aim of the PTBST is to promote high standards in trauma psychotherapy. The current priority for the PTBST is to promote information and education in evidence-based psychotraumatology.

## The situation in Switzerland

There are many psychiatrists and clinical psychotherapists in Switzerland, an increasing proportion of whom are trained in CBT. However, only a minority has acquired special expertise in trauma-focused psychotherapy. During their psychotherapy training, psychiatric residents and clinical psychologists are provided with the basics of evidence-based treatment for trauma-related disorders. At the University of Zurich, experienced psychotherapists can enroll in a 2-year course of “Advanced Studies in Psychotraumatology.”

A special challenge is the treatment of traumatized refugees. Once they have made it to a safe host country, they face a variety of post-migration living difficulties (Schick et al., 2016). They also suffer from emotional dysregulation which makes it even more difficult to deal with daily hassles (Nickerson et al., 2015). As many refugees are not sufficiently proficient in the locally spoken language, psychotherapy needs to be provided with the help of interpreters.

Given the globalization of our world, a good psychotherapist needs to be a culturally sensitive psychotherapist (Schnyder, 2013). On the one hand, it is a privilege to learn from our patients who come from other cultural backgrounds. On the other hand, we should also always reflect on our own individual cultural backgrounds when working as therapists. Europe, due to its cultural diversity, is in a unique position regarding this, providing us with a wealth of cross-cultural experiences.

## The situation in Turkey

Psychotraumatology in Turkey started with studies on domestic violence, sequelae of torture, and psychiatric consequences of childhood trauma with a particular emphasis on dissociative disorders (Şar, Yargiç, & Tutkun, 1996). However, psychotraumatology became mainstream in mental health after the large-scale earthquakes in the Marmara area in 1999. From a cultural point of view, trauma is endemic and almost normative in Turkey compared to Western Europe including the civilian, police, and military exposure to terrorist acts of political motivations with a long history. A most recent challenge to social life in the community has been the open-door policy of the state administration for more than 3 million refugees from Syria who entered the country in a very short period, which exceeds the scope of any examples experienced in Western Europe.

There is no national society of psychotrauma but various societies such as the Psychiatric Association of Turkey and the Turkish Psychologists’ Society have workgroups interested in the subject. In addition to other approaches such as phase-oriented trauma treatment of complex PTSD and dissociation (Şar, 2011), EMDR has become widespread during the last few years and also a national EMDR-society has been founded. Mental health services, including psychotherapy, are covered by state insurance, but psychotherapy (the essential component of trauma treatment) in general is offered within private practice and the patients have to pay themselves. In Turkey, any treatment is legally restricted to the domain of medical practice (psychiatry). Although the number of psychology graduates is growing rapidly, clinical training and certification of this professional group are not well regulated and standardized.

## Discussion

Acknowledgment of trauma and the need for specialized treatment for trauma survivors is growing in the seven European countries. Even with very different histories and sociopolitical situations, the availability of trauma treatments is increasing. National psychotrauma societies are taking an active role in establishing professional standards in psychotraumatology in the countries by developing training curricula and initiating certification of mental health professionals working with trauma. The numbers of mental health care professionals who have been trained to provide evidence-based trauma-focused treatments (BEPP, EMDR, NET, TF-CBT, etc.) are increasing but are not sufficient.

The availability of trauma treatments is closely related to the situation of mental health services. The different sociopolitical situations across countries and socioeconomic discrepancies contribute to the diversities in the development of trauma practice. High-income countries, such as Switzerland, Germany and the Netherlands, have more developed mental health services covered by health care insurance and have many professionals trained in trauma-focused treatment approaches. While in the Eastern and Central European countries, Lithuania or Poland as an example, or Southeastern countries, such as Georgia or Turkey, access to trauma treatment is limited. However, there is still a gap between the need for trauma-focused treatment and the availability of such treatment, especially for the complex cases. The need for trauma treatment for refugees across Europe is a major issue, and as a result, therapists need to learn more cultural sensitivity.

We assume the expert opinions from the seven European countries reflect, to a certain extent, Europe as a whole because these seven countries represent much diversity within Europe. The lack of data about the state of affairs in the different countries is impressive.

The ESTSS has a potential to increase the awareness of trauma-related problems within Europe and needs to stimulate evidence-based trauma treatments. A European-level training curriculum in psychotraumatology could further increase the availability of high-quality trauma-informed care. The *European Certificate in Psychotraumatology* developed by ESTSS could provide a platform for developing standards in training and practice. There is a need for statewide policies in Europe within health care systems to ensure trauma treatment for those in need and for prevention and awareness of traumatic stress within society and in high-risk groups. The ESTSS therefore should set up a European-level project like The European Network for Traumatic Stress (TENTS) for disasters (Witteveen et al., 2012), to develop an expert-informed, data-based systematic knowledge base about the need for and quality of trauma treatment all over Europe which would serve as a blueprint for nationwide policies.
